# Yellow nail syndrome in an elderly sudanese female: A case report

**DOI:** 10.1002/ccr3.5809

**Published:** 2022-05-15

**Authors:** Abdelmuniem Ahmed, Mohamed Y. Yousif, Isam Abdelmageed, Moh. Mah. Fadelallah Eljack, Khabab Abbasher Hussien Mohamed Ahmed, Malaz Tarig AbdAlla Mohamed, Sulieman Abdelkareim G. Mohammed, Elhadi B. Salih, Dina H. Osman

**Affiliations:** ^1^ Physiology department Faculty of Medicine University of Gezira Gezira Sudan; ^2^ Resident Physician Sudanese medical specialisation board Khartoum Sudan; ^3^ Consultant chest physician Hasahesa teaching hospital Gezira Sudan; ^4^ Faculty of Medicine Medical Doctor at Medani Heart Centre University of Bakht Alruda Medani Sudan; ^5^ Faculty of Medicine University of Khartoum Khartoum Sudan; ^6^ Medical officer Federal ministry of health Khartoum Sudan; ^7^ Faculty of Medicine University of Gezira Gezira Sudan

**Keywords:** acute respiratory distress syndrome, ARDS, edema, lymphedema, yellow nail syndrome

## Abstract

Yellow nail syndrome is a rare lymphatic abnormality without clear pathogenesis. Hereby, we report a 70‐year‐old Sudanese female patient who presented with recurrent cough, recurrent lower limb swelling, and yellowish nail discoloration diagnosed as yellow nail syndrome but unfortunately passed away due to acute respiratory distress syndrome (ARDS).

## Abstract 2

The co‐existence of recurrent respiratory affection and non‐pitting edema in patients of more than 40 years should raise suspicion of yellow nail syndrome even in absence of sinusitis, bronchiectasis, or positive family history.

## 1 INTRODUCTION

Yellow nail syndrome is a very rare disorder that has been noticed since 1927.^1^ It affects both sexes equally, with an age of more than 40 years being typical.^2^ The exact pathogenesis remains unclear but lymphatic system anatomical and functional abnormalities remain the predominant theory, while other hypotheses suggest autoimmune, cancer, and paraneoplastic roles.^3^ Also, a very rare familial case has been reported.^1^

Although it is only found in 27%–60% of patients The diagnosis depends on the presence of 2 out; of the characteristic nail changes, respiratory tract infection, and lymphedema. The latter occurs in up to 80% of cases and maybe the first sign.^1^ The common nail changes that have been described include nail discoloration (pale yellow to dark green), nail hyperkeratosis with loss of the lunula, onycholysis, proximal nail‐fold erythema over curvature, cross‐ridging, very hard and difficult‐to‐trim nail, increased nail thickness, and slowed longitudinal growth.^1^, ^3^ Respiratory manifestations that are encountered include pleural effusion, bronchiectasis, chronic cough, and frequent sinusitis.^1^ The condition may resolve spontaneously especially when it is paraneoplastic or cancerous.^1^

Oral vitamin E and fluconazole showed a good response, while intralesional steroids, oral zinc sulfate, and subcutaneous immunoglobulins showed promising data.^1^ Although the prognosis appears to be favorable; it requires extensive research.

## 2 CASE PRESENTATION

A 70‐year Sudanese female, diabetic and hypertensive with a history of ischemic stroke 7 years ago resulted in residual right‐sided weakness; presented complaining of fever and altered level of consciousness for 2 days with a history of recurrent productive cough and recurrent right lower limb swelling for months. There is no family history of a similar condition or connective tissue disease. The patient is neither a smoker nor an alcohol consumer. Her current medications included Glimepiride 4 mg and Losartan 50 mg.

**FIGURE 1:**
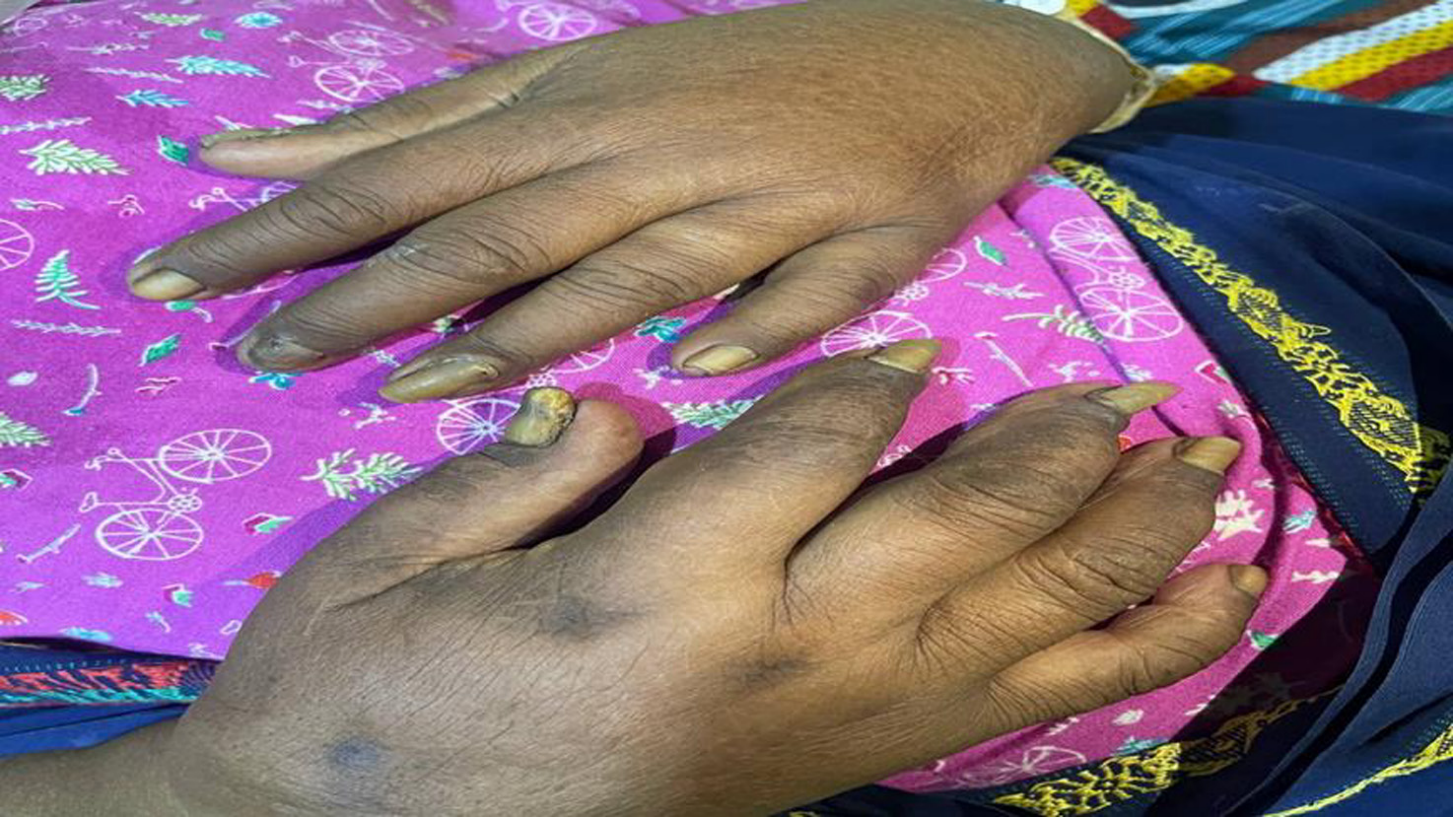
Yellow and thickened fingers’ nails with lymphedema

**FIGURE 2:**
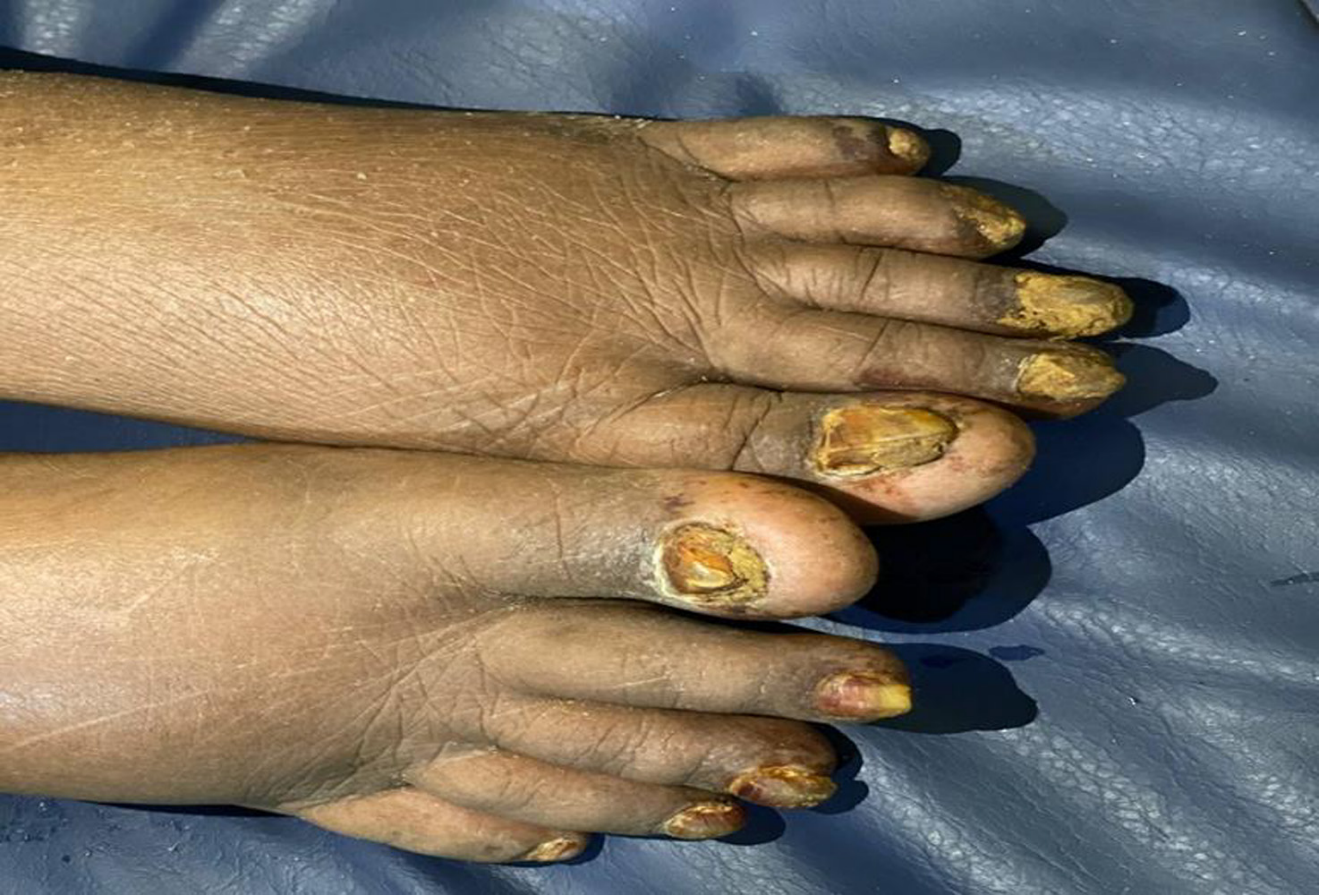
Yellow and thickened toes’ nails with lymphedema

Clinical examination revealed awake, confused patients (GCS 14/15), otherwise clear neurological examination, BP 150/90 mmHg, pulse was 80 beats per minute, SPo2 on 99% of room air. There was yellowish discoloration of her nails in both upper and lower extremities associated with separation from the nail beds (Figures 1 and 2). Chest examination revealed a right‐side stony dullness, decreased air entry, and diminished vocal resonance. There is also a pitting edema in the right lower limb.

**FIGURE 3:**
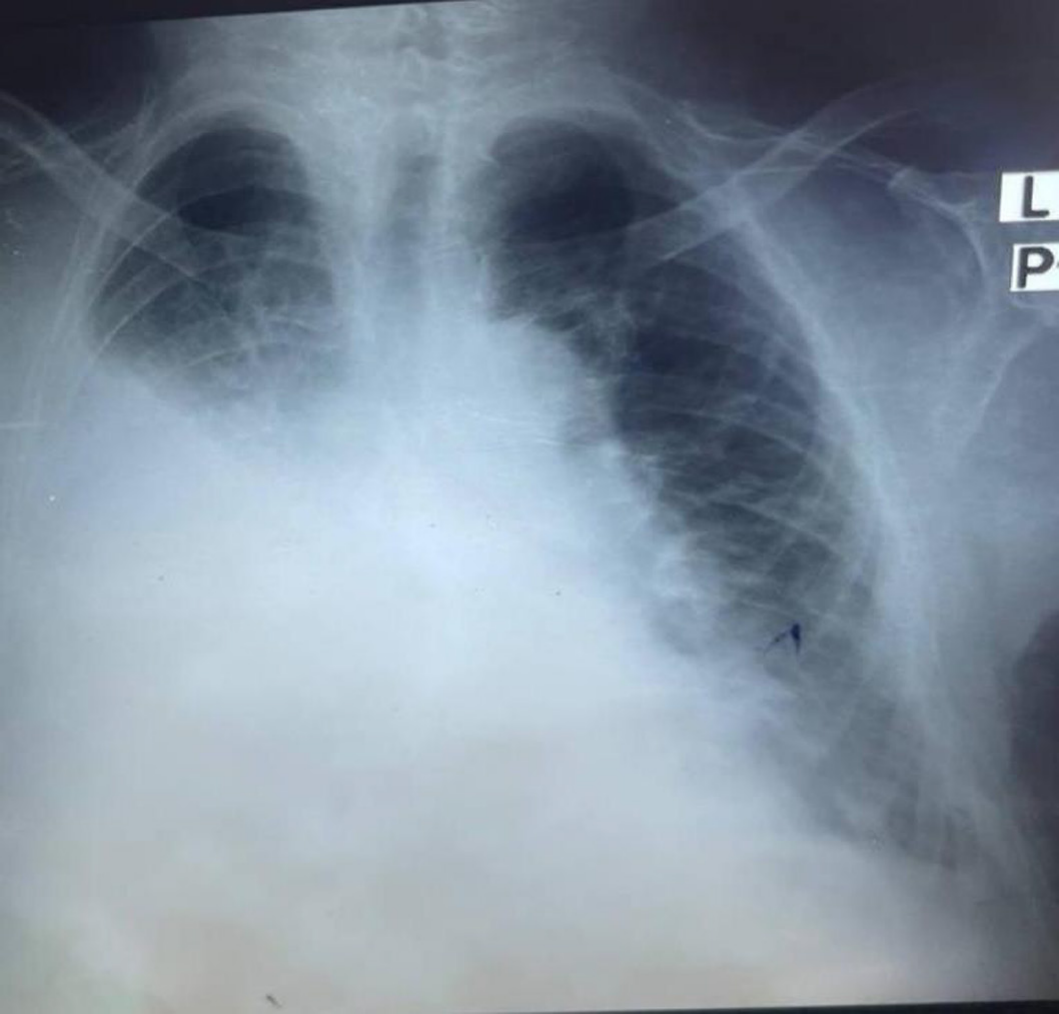
Chest X‐ray revealed a moderate right‐sided pleural effusion

Laboratory investigations showed random blood glucose of 44 mg/dl, positive blood film for malaria, and normal renal and liver function tests. A chest radiograph revealed a moderate right‐sided pleural effusion (Figure 3). Therapeutic thoracentesis was done with 2 liters of a straw‐colored fluid removed, sent for the microscopic examination which showed a hemorrhagic background pleural fluid, that is made of mixed mononuclear inflammatory cells with lymphocytic predominance and it was containing 3.0 g/dl proteins and 57.5 mg/dl glucose.

Diagnosis of yellow nail syndrome with pleural effusion and complicating pneumonia was made.

The patient managed with Quinine infusion of 600 mg T.D.S, 3rd generation cephalosporin, vitamin E, and a prophylactic dose of heparin, however, the condition deteriorate progressively and the patient passed away on the fifth day of admission due to acute respiratory distress syndrome (ARDS).

## 3 DISCUSSION

Yellow nail syndrome was first discovered in 1927, but the first case series of the disease was presented by Samman and White in 1964.^4^ Thirteen cases were presented sharing the common features of thickened yellow nails with growth rates slower than normal (<0.25 mm/week) in comparison with (0.5–1.2 mm\week) in normal individuals.^4^ Lymphedema presented in 10 out of the 13 cases, along with respiratory manifestations consistent with pleural effusion, bronchiectasis, and chronic sinusitis, which are late manifestations of the syndrome. Most cases of YNS are sporadic.^5^ The concept that YNS is an inheritable disorder is based on very little evidence. Despite that, rare familial cases were reported with a doubted autosomal dominant transmission pattern. Very few juvenile cases of the disease were reported.^6^, ^7^ In this section, we discuss the intersections of this patient's YNS presentation with cases reported in the literature.

The presentation of this case is relatively late, as the patient presented at 70 years old. Patients of YNS usually present between their 4th and 6th decade of life.^8^ However, like known cases in the literature,^9^, ^10^ this case presents the classic triad of yellow nail syndrome consisting of yellow and thickened nails (Figures 1 and 2), respiratory manifestations (Figure 3), and lymphedema (Figures 1 and 2), and like other cases of YNS, the case was diagnosed clinically, as Yellow nail syndrome's diagnosis is a clinical one.^2^ And as suggested by Hiller et al, can be made with two of the three known manifestations of the disease, as the observation of these signs may resolve over time.^11^ Nonetheless, a diagnosis of the condition cannot be made in the absence of nail abnormalities.^2^ In addition, hence, based on the look of the patient's nails, chest radiograph, and lower limb swelling, the diagnosis was confirmed.

The case was sporadic. No similar signs of the disease were reported in the patient's family. Patients also presented with ankle and foot edema, as in the majority of the cases reported by Samman and White in their thirteen case series report^9^ and 9 out of 17 cases presented by Bull et al.^12^ No rhinosinusitis was reported in this patient's condition.

The diseases known to be associated with YNS are malignancies, immunodeficiency states, connective tissue diseases, diabetes mellitus, thyroid dysfunction, hemochromatosis, obstructive sleep apnea, Guillain–Barre syndrome, xanthogranulomatous pyelonephritis, tuberculosis, myocardial infarction, nephrotic syndrome, exudative enteropathy, hypoalbuminemia, and drugs (thiol compound therapy).^2^

The therapeutic thoracentesis of the patient showed a straw‐colored fluid of two liters, which can be expected in a pleural effusion patient and is consistent with the patient's presentation with fever, as lower respiratory tract infections, pneumonia, and bronchiectasis are present in half of YNS patients.^2^, ^13^ In addition, hence as indicated by her symptoms, the patient had pneumonia.

Generally, analysis of pleural fluid of YNS patients is characterized by being transudative, with high protein content.^2^, ^14^ Analysis of this patient's pleural fluid showed mixed mononuclear inflammatory cells with lymphocytic predominance. With 3.0 g/dl protein and 57.5 mg/dl glucose content, previous findings in the literature entail that lymphocytic predominance is the rule when it comes to cellular analysis.^8^ The patient's pleural fluid is also exudative by protein criteria and transudate by cholesterol and lactate dehydrogenase criteria as with previously known cases in Maldonaldo et al in which he performed high yield analysis of pleural fluid of 41 patients.^8^

The patient's chest radiograph shows moderate right‐sided pleural effusion (Figure 3). This radiographic finding is particularly similar to a case reported by Maldonaldo et al where the patient's pleural effusion was bilateral but particularly larger in the right chest.^2^

Cases of YNS’s respiratory manifestations are treated with postural drainage, other pulmonary hygiene measures, and antimicrobials.^2^ Treatment of pleural effusion depends on the severity of the effusion. Therapeutic thoracentesis might suffice in management. With pleurodesis coming as an option for recurrent effusion patients,^2^ the use of topical steroids or vitamin E has been described through the evidence supporting their use remains scarce.^8^, ^15^

No definitive treatment for YNS was confirmed until now.

However, several treatment regimens for the disease were proposed in the literature, but they remain as treatment efforts for individual cases. Fluconazole, vitamin E, and topical steroids have been the most popularly used treatments for YNS, but their ascribed treatment properties are still under question due to the scarcity of supportive evidence.^8^, ^15^, ^16^

Like many other cases with the disease,^2^ this case was managed with supportive treatments to ameliorate the severity of the symptoms. Specifically, regarding the patient's treatment for YNS, along with the therapeutic thoracentesis, she was treated with vitamin E. The patient took Glimepiride 4 mg and Losartan 50 mg. Quinine for malaria, 3rd generation cephalosporin for pneumonia, and a dosage of heparin for hypertension prophylaxis were prescribed for her diabetes and hypertension. Despite efforts, the patient's condition deteriorated quickly and she passed away due to an episode of acute respiratory distress syndrome (ARDS). Up to our knowledge, there is no published literature linking yellow nail syndrome to a specific fatal complication.

The course of the disease is generally known to be benign; the prognosis of yellow nail syndrome cases shows decreased life longevity in comparison with the control population.^2^ Although when presented to the clinic the patient underwent the proper investigations to diagnose the syndrome and was managed with the up‐to‐date known management of the disease, this late presentation and hence identification of the patient's condition might have affected her prognosis, along with the patient's co‐morbidities and age which might have further complicated the condition and our understanding of the progression of the case.

## 4 CONCLUSION

Yellow nail syndrome is a syndrome that presents with yellow thickened nails, respiratory manifestations, and lymphedema. In this case, the patient has presented in the 8th decade of her life as a sporadic case with the classic triad of the disease with no complications. Patients’ pleural profile and chest radiograph matched that of patients in the existing literature. Also, the case had a history of hypertension, diabetes, and stroke. Although diabetes has been noted to be associated with yellow nail syndrome, there is no conclusive evidence regarding its relation to hypertension despite several noted cases until now. The patient passed away after an episode of acute respiratory distress syndrome (ARDS). No similar reports of this specific case of death were found in the literature.

## AUTHOR CONTRIBUTIONS

AA, MYY, and IA took history, did investigations, and participated in writing the first draft. MMF, KAH, and MTA wrote first and final drafts. SAG, EBS, and DHO wrote and revised the final draft. All authors contributed significantly to the study.

## CONFLICT OF INTERESTS

The authors have no conflict of interest to declare.

### CONSENT TO PARTICIPATE

Firstly, verbal and written consent to participate and to publish this information was obtained from the patient; then after she passed away, it was obtained again from her relatives.

### CONSENT TO PUBLISH

Consent for publication was obtained from all authors.

## Data Availability

The datasets used and\or analyzed during the current study are available from the corresponding author upon reasonable request.
